# Increased functional connectivity of motor regions and dorsolateral prefrontal cortex in musicians with focal hand dystonia

**DOI:** 10.1007/s00415-025-13018-y

**Published:** 2025-03-22

**Authors:** Stine Alpheis, Christopher Sinke, Julian Burek, Tillmann H. C. Krüger, Eckart Altenmüller, Daniel S. Scholz

**Affiliations:** 1 Institute of Music Physiology and Musicians’ Medicine, Drama and Media, Hannover University of Music, 30175 Hannover, Germany; 2 Department of Musicians’ Health, University of Music Lübeck, 23552 Lübeck, Germany; 3 Institute of Medical Psychology, University of Lübeck, 23562 Lübeck, Germany; 4 Department of Psychiatry, Social Psychiatry and Psychotherapy, Hannover Medical School, 30625 Hannover, Germany; 5 Hannover Medical School, 30625 Hannover, Germany; 6 Department of Psychology, University of Lübeck, 23562 Lübeck, Germany; 7 Center for Systems Neuroscience, Hannover, Germany

**Keywords:** Musician’s dystonia, Resting-state fMRI, Functional connectivity, Basal ganglia, Motor networks, Adverse childhood experiences

## Abstract

**Background:**

Musician’s dystonia is the most common form of focal task-specific dystonia and is suggested to be the result of dysfunctional communication among sensory-motor networks. Thus far, few functional connectivity studies have investigated musician’s dystonia specifically, leaving its exact pathophysiological mechanisms unclear. The goal of this study was to verify connectivity findings from other task-specific dystonias on a large sample of musician’s hand dystonia patients and to analyze associations with possible adverse childhood experiences, a suggested risk factor for dystonia.

**Methods:**

Forty professional musicians suffering from musician’s hand dystonia and a matched control group of healthy musicians underwent resting-state functional magnetic resonance imaging and answered the childhood trauma questionnaire. Using a seed-to-whole brain approach, functional connectivity alterations between motor cortices, the prefrontal cortex, the basal ganglia and the thalamus were analyzed.

**Results:**

Musician’s dystonia patients showed increased functional connectivity of the dorsolateral prefrontal cortex with the putamen and the pallidum, especially in right-side affected patients. Patients further displayed increased connectivity of the left thalamus and the right lateral premotor cortex. No associations between functional connectivity, duration of disorder and childhood adversity were observed.

**Conclusion:**

The findings are consistent with previous research, highlighting the pathophysiological importance of the basal ganglia. Altered resting-state functional connectivity may reflect underlying neuroplastic changes in musicians with dystonia that lead to an altered flow of information, disrupting movement inhibition. Involvement of the dorsolateral prefrontal and premotor cortices further suggests that motor disturbances occur in the early planning phase of a movement. The findings indicate that a holistic re-training approach with and without the instrument could be beneficial for regaining motor control.

**Supplementary Information:**

The online version contains supplementary material available at 10.1007/s00415-025-13018-y.

## Introduction

Focal task-specific dystonia (FTSD) is characterized by diminished motor control and muscular co-contractions during the execution of highly trained movements [1]. In its most prominent form it affects professional musicians, who experience a lack of control, e.g., involuntary flexion or extension of the fingers while playing their instrument [2]. Thus far, only a few studies have investigated functional connectivity alterations in musician’s dystonia (MD) exclusively, which is, therefore, the objective of this study.

Throughout the last decade, idiopathic focal dystonia has been increasingly identified as a network disorder where higher-order motor control systems communicate dysfunctionally [3–10]. Together with (controversially discussed) maladaptive neuroplasticity [11–13], altered network communication is suggested to be the reason for the observed loss of surround inhibition and abnormal sensorimotor processing causing dystonic movements [14]. Musician’s dystonia is considered a task-specific focal dystonia, meaning symptoms are restricted to one area and one certain task. It occurs either in the limbs (hands, fingers, and feet) or in the muscles of the mouth and tongue (embouchure dystonia). Other task-specific dystonias are writer’s cramp (WC) or yips in golfers. If not injury-induced, it mostly occurs idiopathic, i.e., without a known cause. Other forms of idiopathic non-task-specific focal dystonias are, e.g., cervical dystonia and blepharospasm. In all forms of focal dystonia, alterations in the cortico-basal ganglia-thalamo-cortical circuit [15] and the cortico-cerebellar-cortical circuit [16–19] are frequently reported. While the basal ganglia are involved in motor planning and the inhibition of undesired movements [20], the cerebellum is responsible for the fine-tuning and sensory prediction of movements [21, 22]. Thus, alterations of functional connectivity (FC) in these areas would likely lead to the observed uncoordinated motor movements, loss of control, and involuntary co-contractions. While the mechanisms behind the task-specificity of musician’s dystonia are not fully understood, it is suggested to result from increased involvement of the cerebellum, the thalamus, and motor cortical regions, which appear altered during the management of motor execution [23]. Furthermore, altered proprioceptive and tactile feedback is suggested to influence voluntary motor control [24]. Resting-state (rs) fMRI offers the possibility to investigate FC without a given task, making it a useful tool when investigating network communication in different forms of dystonia. The technique correlates spontaneous fluctuations in blood oxygen level-dependent signals between brain areas [25, 26] with the idea that areas communicating with each other synchronize their activity. Recent literature reviews [4, 9] on FC alterations during resting state among patients with focal dystonia report decreased or increased FC to be most commonly found in the precentral gyrus, the postcentral gyrus, the right supplementary motor area (SMA), and the left putamen. In patients with FTSD (e.g., writer’s cramp, WC), both increased and reduced FC were further observed in the sensorimotor network [1, 27], as well as in cerebellum-cortical and basal ganglia-cortical networks [28]. However, while all focal dystonias seem to share alterations in these networks, task-specific dystonias appear to present more complex cortical abnormalities than non-task-specific dystonias [3]. Furthermore, structural alterations are observed especially in directly task-related areas in FTSD, while mostly cerebellar alterations are found in non-task-specific dystonia patients [29].

While several studies have investigated blepharospasm, cervical dystonia, or writer’s cramp, there are, to our knowledge, only two studies focusing solely on resting-state FC in musician’s dystonia. One study investigating embouchure dystonia found increased FC of the secondary somatosensory cortex and the mouth area of the sensorimotor cortex with the lateral motor-function network [30]. Another study on 21 pianists and 34 healthy controls observed increased FC of the anterior right putamen with the basal ganglia resting-state network [8]. While the first study found no correlation between FC alterations and the severity of dystonia, the second study reports an association between putaminal basal ganglia FC and musical skill in MD patients. Other comparable studies on writer’s cramp reported reduced FC between the somatosensory cortex (S1), the primary motor cortex (M1), and the dorsolateral prefrontal cortex (dlPFC), as well as altered FC in the cerebello-basal ganglia-thalamo-cortical circuit [31] and reduced coupling of premotor-parietal regions [32]. Given the pathophysiological differences between MD and WC [33], the goal of this study was to verify hitherto made findings in the context of musician’s dystonia and possibly reproduce them within a large sample of MD patients affected in the hand. Based on the above-presented literature, we especially focused on the M1, S1, SMA, premotor cortex (PM), dlPFC, and the basal ganglia, namely the pallidum and the putamen.

Recent studies by our group and others have additionally focused on the psychological risk factors of dystonia, investigating stress reactivity and childhood adversities since they have repeatedly been reported by patients [34]. In these studies, slightly higher rates/severity of adverse childhood experiences (ACEs) were observed [35, 36], which is usually linked to alterations in the amygdala, hippocampus, and prefrontal cortex [37]. Stress associated with ACEs has been found to affect the functional interaction between the hippocampus and striato-cortical areas, which in turn has a negative impact on motor memory consolidation [38]. It furthermore appears to affect FC in the default mode network, fronto-parietal network, salience network, and central executive network [37, 39]. Investigating whether these alterations directly impact neurological alterations observed in dystonia was the second goal of this study.

Building on this recent line of investigation, we included an assessment of childhood trauma in our analysis to explore the involvement of childhood adversity in the pathophysiology of dystonia. Based on the above-presented findings, we hypothesized that MD patients show altered functional connectivity within the cortico-basal-ganglia-thalamo-cortical circuit and that these alterations in functional connectivity are associated with adverse childhood experiences in musicians.

## Methods

### Participants

This paper is part of a larger project investigating risk factors for focal dystonia in musicians [36]. A total of 40 MD patients (all affected in the hand or fingers) and 39 healthy musicians participated in the study (Table 1). Healthy musicians (HM) were matched to the patients by age, instrument and sex as best as possible to form the control group. Patients were recruited from the patient data base of the Institute of Music Physiology and Musicians’ Medicine in Hannover, where all of them were diagnosed with focal dystonia by experienced experts. The controls were recruited via local symphony orchestras, the local university of music, an open call on social media and through personal contacts of the participating patients. Exclusion criteria were injury-induced dystonia, a history of major medical or neurological problems, current severe psychological disorders, pregnancy and insufficient eyesight. An additional exclusion criterion for healthy musicians was any kind of movement disorder. Since some MD patients receive medication and an exclusion would have reduced the participant numbers drastically, current medication was not an exclusion criterion but trihexyphenidyl and psychotropic medication were controlled for in the analysis. For the study conducted at the Hannover Medical School between April 2022 and April 2023, all participants provided informed consent and were ensured complete anonymity and confidentiality. Additionally, they received full travel reimbursement and a compensation of 60€.

**Table 1:** Descriptive characteristics of participants

Parameter	MD (*n* = 37)	HM (*n* = 36)	Test statistic	*p*-value
Age in years* M (*± *SD)*	47.0 (9.86)	48.2 (11.9)	t(71) = − 0.468	0.641
Sex: male/female/other *n*	23/14/0	23/13/0	χ^2^(1, *n* = 73) = 0.023	0.879
Affected hand				
Right side *n*	16	–		
Left side *n*	21	–		
Handedness: right/left/both-handed/na	34/2/0/2	30/4/0/2	χ^2^(1, *n* = 70) = 0.860	0.354
Dystonia onset: age in years *M (*± *SD)*	34.2 (9.86)	–		
Participants receiving medication *n (%)*	14 (37.8%)	10 (27.8%)	χ^2^(1, *n* = 73) = 0.837	0.360
Trihexyphenidyl	5 (13.5%)	0	χ^2^(1, *n* = 73) = 5.22	0.022*
Psychotropic medication	1 (2.7%)	1 (2.8%)	χ^2^(1, *n* = 73) = 0.00	0.984
Other	8 (21.6%)	9 (2.5%)	χ^2^(1, *n* = 79) = 0.12	0.733
CTQ total score *Mdn (IQR)*	34 (16)	29.5 (9.75)	W = 490	0.052

Test statistics *W* indicates the Wilcoxon rank-sum test

*Mdn* Median, *IQR* Interquartile Range, *CTQ* Childhood Trauma Questionnaire, *MD* musicians’ dystonia patients, *HM* healthy musicians

^*^p < 0.05

Of the 79 participants who completed fMRI scanning, one person had to be excluded because of incidental medical findings, one person because of a panic attack during the MRI scan and four participants because of excessive head motion resulting in less than 120 of 465 volumes after head-motion scrubbing. This left 73 participants (MD = 37; HM = 36) to be included in the final analysis.

### Experimental design

All participants completed structural T1-weighted MRI scanning first, followed by rsfMRI. Additionally, diffusion tensor imaging and an experimental task followed these scans which are not part of the current study and are reported elsewhere [36]. During functional scans, the participants were asked to lie still with open eyes, fixating a cross on a screen. Following the fMRI, the participants gave demographic information about disorder duration and family history and answered the Childhood Trauma Questionnaire (CTQ) [40]. The CTQ assesses adverse childhood experiences with 28 items, measuring emotional and physical neglect, as well as emotional, physical, and sexual abuse. Answers are given on a 5-point Likert scale and the questionnaires were administered in the German [41] or English versions.

### Data-acquisition and preprocessing

All scans were conducted on a Siemens 3 T Skyra MRI scanner (Siemens Healthineers, Erlangen, Germany) with a 64-channel head coil. The following parameters were used to acquire T1-weighted images: number of slices = 176; voxel size = 1 × 1 × 1 mm; repetition time (TR) = 2500 ms; echo time (TE) = 2.9 ms; field of view (FoV) = 256 × 256 × 176 mm; and flip angle = 8 degrees. For the resting-state fMRI images, the following echoplanar imaging (EPI) sensitive sequence was used: voxel size = 2 × 2 × 2 mm, FoV = 208 × 208 × 156 mm, multiband acceleration factor = 6, TR = 1310 ms, TE = 36.0 ms, and 470 volumes.

The preprocessing was performed using the Data Processing Assistant for Resting-State fMRI (DPARSF, Yan and Zang 2010, http://rfmri.org/DPARSF), which is based on Statistical Parametric Mapping (SPM, http://www.fil.ion.ucl.ac.uk/spm) and the toolbox for Data Processing & Analysis of Brain Imaging (DPABI, Yan et al. 2016, http://rfmri.org/DPABI) in MATLAB (version 2024a). After removing the first five volumes of the functional data to account for initial signal instability, the images underwent slice-time correction and were realigned to a mean image. Voxel-specific head motion parameters were calculated, followed by co-registration of the structural images to the functional images and segmentation into white matter (WM), grey matter (GM) and cerebrospinal fluid (CSF) using DARTEL. Head motion parameters and nuisance covariates (global signal, WM and CSF) were regressed out [42]. This was followed by the normalization of the images to MNI space (using DARTEL) and temporal bandpass filtering (0.01–0.08 Hz). Afterwards, framewise displacement (FD) was calculated and those time points showing FD of more than 0.5 mm were removed using head motion scrubbing [43]. The images were then smoothed with a Gaussian kernel of 4 × 4 × 4 mm (full width at half maximum).

### Seed definition and functional connectivity analysis

**Fig. 1:**
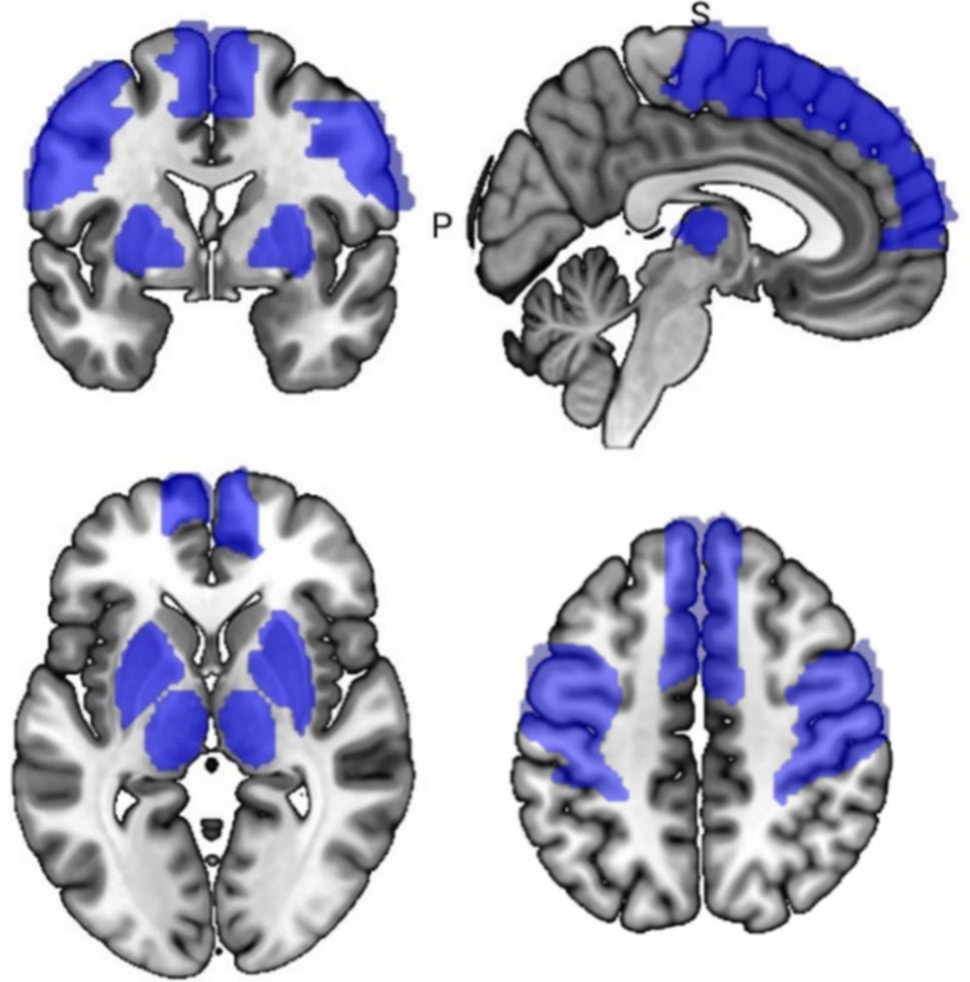
Visualized mask of the regions of interest chosen as seed regions: dorsolateral prefrontal cortex, supplementary motor area, precentral gyrus, postcentral gyrus, thalamus, pallidum and putamen. Coronal and axial slices are presented in neurological convention (left = left hemisphere, right = right hemisphere). Centered at *x* = 4, *y* = 0, *z* = 0 and *z* = 46). Visualized with MRIcroGL

After preprocessing, functional connectivity analysis was conducted with the DPARSFA toolbox in form of a seed-to-whole brain analysis. Based on the literature presented above, our focus was on regions belonging to the cortico-basal ganglia-thalamo-cortical loop. Accordingly, the following seven regions of interest (ROIs) were selected: primary motor cortex (precentral gyrus), dorsolateral prefrontal cortex (superior frontal gyrus, medial), supplementary motor area, somatosensory cortex (postcentral gyrus), thalamus, putamen and globus pallidus. These areas were included into the analysis as defined by the automated anatomical labelling atlas 3 (AAL3) [44], except for the thalamus, which was based on the AAL [45]. The entire regions were included as visualized in Fig. 1. All regions were included bilaterally, resulting in 14 ROIs. Due to the inconclusive findings regarding resting-state FC alterations in task-specific dystonia patients [4], the cerebellum was not selected as seed area but only included in the whole-brain analysis. Using Pearson’s correlation, the averaged time course of all voxels within each region was correlated with the time course of all other voxels. The resulting individual correlation values were subsequently normalized using Fisher’s *z* transformation for further analysis in a second level model.

### Statistical analysis

The second level analysis was performed in SPM12 (SPM12; Wellcome Department of Imaging Neuroscience, London, U.K.). Two-sample t-tests were used to determine differences in functional connectivity between MD patients and healthy musicians. These differences were assessed in separate general linear models (GLM) for each seed region of interest, including age, sex, and medication (psychotropic medication: 1 MD, 1 HM participant; trihexyphenidyl: 5 MD participants) as covariates of no interest. Two contrasts (MD > HM and MD < HM) were calculated to estimate increased or reduced FC in the MD group compared to the HM group. Family-wise error (FWE) correction at cluster level was applied to account for the number of voxels analyzed in the seed-to-whole brain approach. We further applied Bonferroni correction to account for multiple comparison of 14 different ROIs, resulting in a significant threshold for each seed region of p_FWE_ < 0.05/14 = 0.00357. The anatomical locations of the significant clusters were defined using the AAL3 atlas.

#### Analyses of psychological parameters

Further statistical analyses were conducted in R (Version 4.3.1) and jamovi (Version 2.3). ROI analysis revealed significant FC differences for four regions. Correlation estimates averaged across the significant clusters were extracted from SPM and added into a linear regression model together with the CTQ total scores to assess whether adverse childhood experiences predict FC changes in musicians. All four models were controlled for additional effects of handedness. To further explore what drives functional connectivity in MD patients, additional regression analyses were conducted with only the MD group. In these models, duration of the disorder was selected as predictor to assess whether the FC between identified areas is dependent on the duration patients have suffered from dystonia. Additionally, the affected hand was added into the regression as a factor, to assess possible effects of lateralization. Excluding the above-named participants, 16 MD patients were left in the “right-side affected” group and 21 MD patients in the “left-sided affected” group.

## Results

### Demographic data

As the groups were purposefully matched, MD patients and HM did not differ in their age or sex distribution. Table 1 summarizes the demographic data of the participants, which has partly already been described in a previous paper [36].

### Increased functional connectivity

For the contrast MD > HM, four regions were identified that showed increased functional connectivity among MD patients with one of the seed regions (Table 2). No regions showing decreased functional connectivity (MD < HM) were found.

**Table 2:** Increased functional connectivity in MD patients compared to healthy musicians (MD > HM)

Seed ROI	MNI					
Target regions	*x*	*y*	*z*	Cluster size *k*	*t* value	*p* value
Thalamus L						
Middle frontal gyrus R (56%) and precentral gyrus R (24%)	42	8	38	166	3.94	0.002
dlPFC L						
Pallidum L	− 20	4	2	220	4.76	0.000
Putamen R	26	6	0	144	4.46	0.003
dlPFC R						
Pallidum L	− 18	2	2	152	4.48	0.002

Results are presented FWE_corr_ at cluster level *p* < 0.05 and corrected for multiple comparison of 14 ROIs using Bonferroni correction. Significant clusters were identified based on the AAL3 in MRIcroGL, reporting the percentage of clusters in the respective regions

*ROI* Regions of Interest, *L* left, *R* right, *dlPFC* dorsolateral prefrontal cortex, *MNI* Montreal National Institute of Health Coordinates, *k* number of voxels in the significant cluster

**Fig. 2:**
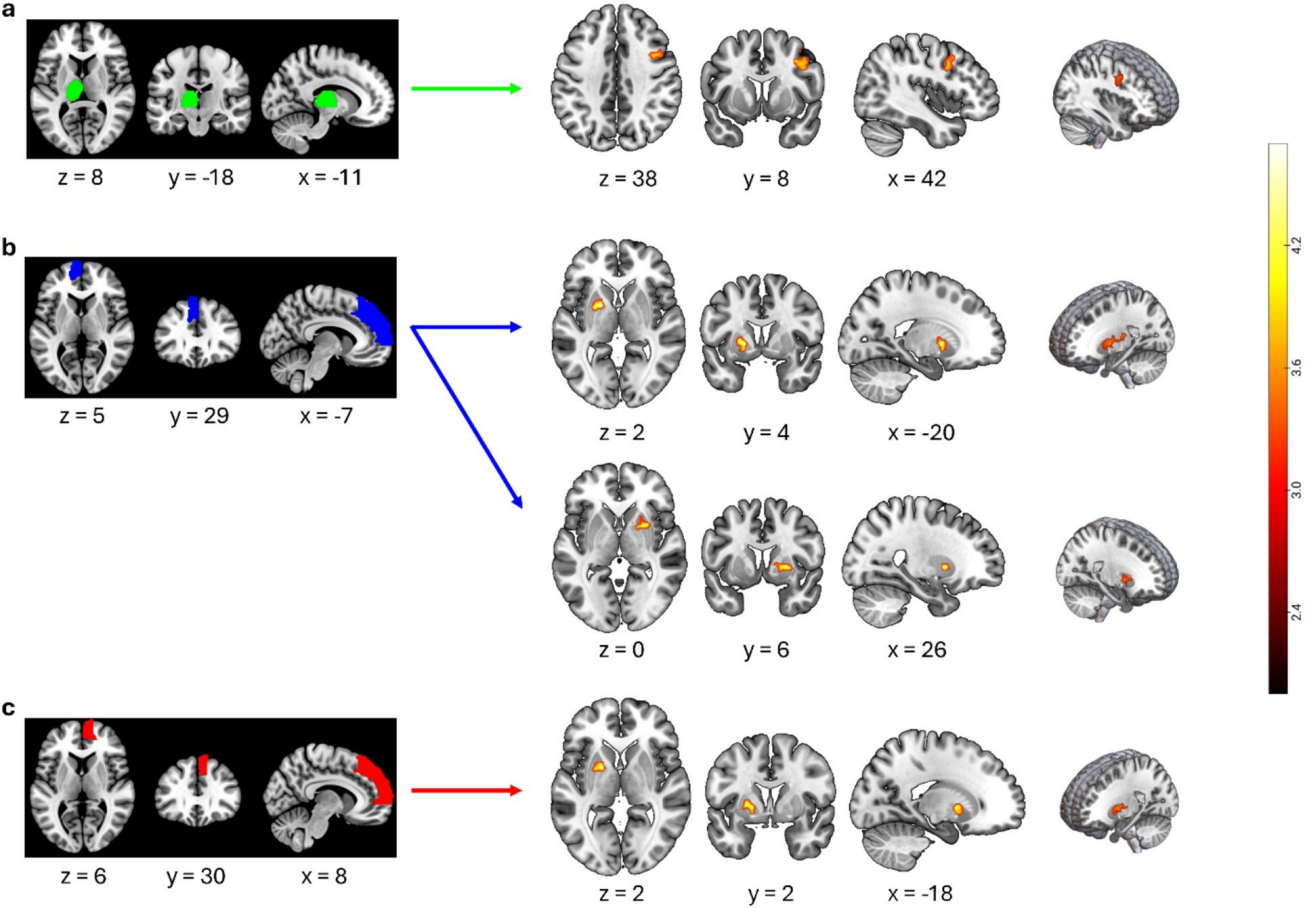
Increased resting-state functional connectivity among MD patients compared to healthy musicians in three seed regions. Seed regions as defined by the AAL/AAL3 are portrayed on the left side: Left thalamus (**a**), left dorsolateral prefrontal cortex (**b**), right dorsolateral prefrontal cortex (**c**). FC maps representing areas showing significantly increased FC (FWE-corrected at cluster level, *p* < 0.004) with the corresponding seed regions are portrayed on the right side. Contrasts are displayed as t-values, indicated by the color bar. The images were visualized using MRIcroGL

A significantly higher resting-state FC was detected between the left-sided thalamus and the right middle frontal gyrus in MD patients compared to healthy controls (Fig. 2a and Table 2). To assess the sensorimotor area the observed cluster in the middle frontal gyrus belongs to, the Human Motor Area Template [46] was used, revealing the peak of the cluster to be located in the lateral premotor cortex, more precisely the right ventral premotor cortex (PMv), extending to the dorsal premotor cortex (PMd). There was further found significantly increased FC in MD patients between the left and right dlPFC and the left-sided pallidum, as well as the left dlPFC and the right-sided putamen (Fig. 2b and c and Table 2). No differences in FC were observed for any of the other seed regions.

Since the relatively large number of MD patients in the current sample offers a unique opportunity, an additional exploratory whole-brain analysis was conducted where further areas und sub-areas of the AAL3 atlas were correlated to the whole-brain (see supplementary material). This is meant to offer further impulses for future research without submitting the analysis to researchers’ bias since only reporting “expected findings” might overlook other relevant areas. In the exploratory whole-brain analysis, healthy musicians showed increased FC between right middle temporal pole and left crus II of the cerebellum. Additionally, further FC alterations between frontal regions, the basal ganglia and the thalamus were found in MD patients. Results were FWE-corrected at cluster level with a significance level of *p* < 0.05, but not corrected for multiple comparisons.

### Regression analyses

#### Associations between FC and childhood adversity

For each area showing increased FC, a separate multiple regression model was built including childhood trauma and handedness (dummy coded) as predictors. All models were tested for linearity, normality of residuals, homoscedasticity, and independence of errors and fulfilled the required model assumptions. None of the models were significant, thereby revealing no association between CTQ and FC in the observed areas.

#### Associations between FC, duration of disorder and affected hand

In the regression analyses among only MD patients, the affected hand was found to be a significant predictor of the FC between the left dlPFC and the right putamen (Table 3) with the model showing overall significance (*F* (2, 34) = 5.05, *p* = 0.012).

**Table 3:** Results of the multiple regression analyses among MD patients

Model					95% CI	
Variable	*B*	*SE*	*t*	*p*	Lower	Higher
dlPFC L – Putamen R						
Intercept	0.49	0.05	10.07	0.000***	0.39	0.59
Duration	0.00	0.00	− 1.06	0.298	− 0.01	0.00
Affected Hand	− 0.13	0.05	− 2.53	0.016*	− 0.24	− 0.03
* R*^2^	0.18					

Multiple regression models explored whether duration of the disorder and affected hand predict FC. Reference group for affected hand was the right hand (right hand = 1, left hand = 0)

*dlPFC* dorsolateral prefrontal cortex, *L* left, *R* right, *B* beta estimates, *SE* standard error, *t* t-value, *CI* confidence interval

^*^p < 0.05; **p < 0.01; ***p < 0.001

**Fig. 3:**
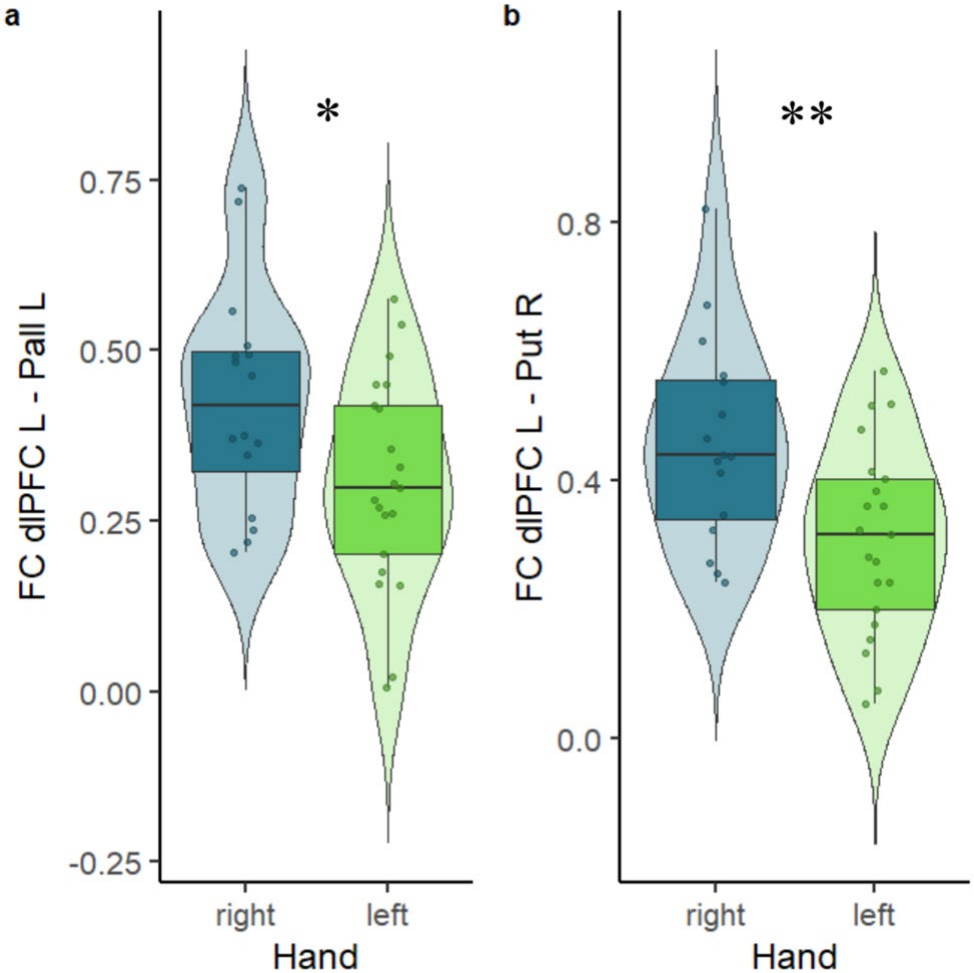
The two plots show significant differences in functional connectivity (FC) of musician’s dystonia patients between the left dorsolateral prefrontal cortex and the left pallidum (**a**), as well as the left dorsolateral prefrontal cortex and the right putamen (**b**) divided by affected hand. Right-side affected patients are displayed in blue, left-side affected patients are displayed in green. * *p* < 0.05; ** *p* < 0.01

None of the other models were significant and duration of the disorder had no effect on functional connectivity in any of the models. Additional analysis using t-test on the FC revealed significant differences between right-side and left-side affected patients in the FC of left pallidum and left dlPFC (*t* (35) = 2.31, *p* = 0.027) and the FC of right putamen and left dlPFC (*t* (35) = 2.99, *p* = 0.005). Differences between right-side and left-side affected patients are visualized in Fig. 3.

## Discussion

This study aimed to confirm and further explore alterations in the functional connectivity of musicians with focal dystonia using resting-state fMRI. Following previous findings [8, 9, 31, 32], we expected alterations in areas belonging to the cortico-basal ganglia-thalamo-cortical circuit as well as further areas responsible for motor movement and executive functioning, more precisely, the primary motor cortex, the somatosensory motor cortex, the SMA, the premotor cortex, the dorsolateral prefrontal cortex, and the cerebellum. Simultaneously, adverse childhood experiences of the musicians were considered since recent findings suggested an involvement of ACE in the development of dystonia, possibly via dysfunctional neurological coping mechanisms. Our findings show increased FC of the thalamus and the premotor cortex, as well as the dlPFC and the basal ganglia (pallidum and putamen), which is consistent with previous studies. However, FC alterations were not associated with the intensity of adverse childhood experiences, questioning the influence ACEs have on the motor circuit alterations of MD patients. FC was furthermore not associated with the duration of the disorder.

### General resting-state functional connectivity

The basal ganglia have long since been at the center of attention of dystonia research. In the cortico-basal ganglia-thalamo cortical circuit, they are crucially involved in the selection and facilitation of desired movements as well as the inhibition of unwanted movements by receiving information from the cortex and projecting back to it via the thalamus [20]. The external segment (GPe) of the pallidum (globus pallidus) is considered an intrinsic nucleus, relaying information for the facilitation of movements, while the internal segment of the globus pallidus (GPi) is categorized as an output nucleus and has an inhibitory function. The putamen, as part of the striatum, is categorized as an input nucleus, receiving information and projections from the cortex [20]. Our findings reflect alterations in the resting-state functional connectivity of each of these nuclei. Even though some studies on focal dystonia report no FC alterations [1] or decreased FC of the putamen [47] and the pallidum [31], several previous studies also observed increased functional connectivity within the basal ganglia network or between the basal ganglia and cortical motor areas [8, 27, 48]. The present findings are, therefore, largely consistent with other studies investigating task-specific dystonia in the resting state. Network approaches found increased functional connectivity of the putamen and the default mode network in a sample of writer’s cramp patients [27] and increased FC of the putamen with the basal ganglia network in MD patients [8]. The differences between previously observed increased and decreased FC might be attributed to methodological approaches or to possible differences in the pathophysiology of WC and MD. While similar in their symptoms, the two disorders manifest during different tasks and in different parts of the hand. In a study of the structural connectome of idiopathic dystonia, increased hub formation of the basal ganglia was observed, as well as a correlation between hub alterations of the pallidum and the severity of writer’s cramp [6]. Even though our findings do not differentiate between GPi and GPe, it is plausible that the observed altered pallidal activity is primarily responsible for the reduced inhibition of dystonia patients. The inhibitory mechanisms relayed through the GPe and the GPi to the thalamus and the motor cortex would be disturbed, resulting in the observed co-contraction of neighboring muscles at the instrument. An overall hyperactivity of the basal ganglia and their motor pathways has been hypothesized before since the basal ganglia have also been found to be overly active in a tactile stimulation task in WC patients [49] and showed abnormal dopaminergic functioning [15]. Since rsfMRI measures synchronization of spontaneous BOLD fluctuations, however, there is no direct inference to be made from our findings to the functional consequences of the observed FC alterations. One possible result of altered resting-state activity of the putamen, which plays an inhibitory role in the motor circuit to enable smooth movement, are dysfunctional projections to the pallidum, which in turn affects motor inhibition when the information is transferred back to the thalamus and the cortex. This would be consistent with findings made during a tapping task, where a stronger inhibitory influence from the globus pallidus to the primary motor cortex was observed in healthy participants compared to WC patients [48]. Simultaneously, the facilitating influence from the globus pallidus back to the cortex was found to be less strong in WC patients. Altered FC might further be an indicator of altered striatal dopamine homeostasis, as activity in the globus pallidus is regulated via dopamine from the substantia nigra [50]. Subsequently, the balance between the globus pallidus and the putamen might be affected by an altered dopamine transmission. Altered striatal dopamine homeostasis has been associated with idiopathic dystonia and is suggested to present a predisposition for developing dystonia [51].

Intriguingly and contrary to other studies, the basal ganglia were functionally stronger connected with the bilateral dlPFC and not with the motor cortex. While other studies observed decreased FC of the basal ganglia and the S1 and the M1 [3, 31, 47] or reduced intracortical FC between M1, SMA, and premotor cortex [30–32], no alterations in these areas were observed in our study. However, as was discussed before [27], while FC alterations might be found in task-based fMRI, which displays activity, our results depict functional connectivity in the resting state only. An absence of motor cortical alterations might, therefore, be the result of musician’s dystonia patients being asymptomatic at rest. Furthermore, none of these studies focused on MD patients, except for one, which investigated embouchure dystonia, which probably differs from musician’s focal hand dystonia in its pathophysiology. Our findings functionally connect the basal ganglia and dlPFC. The dlPFC has been less prominent in dystonia research and has thus far not been associated with basal ganglia dysfunction among dystonia patients but with reduced connectivity with cortical motor areas [31]. In the same study, however, functional connectivity of motor areas and the dlPFC was also found to be increased with increasing duration of WC. Even though the dlPFC is not usually considered part of the motor circuit, it shares a connectivity pattern with the basal ganglia and the thalamus, indicating its central role in the conscious execution of movements [52]. It further appears to play a role in motor learning and selection of movements [53] and has been associated with neurological movement disorders and dystonia, such as “freezing of gait” in Parkinson’s patients [54] or during joystick movements in a sample of torsion dystonia patients [55]. Interestingly, the dlPFC is otherwise mostly associated with psychogenic movement disorders. The research suggests that alterations of a circuit consisting of the prefrontal cortex, the basal ganglia and the thalamus are responsible for an active, albeit unconscious, inhibition of the preparation and execution of movements in patients with psychogenic movement disorders [56, 57]. It could, furthermore, indicate the increased cognitive effort of patients to produce or inhibit certain movements [58], thereby being a mechanism of adaption rather than a disorder contributing factor. Although dystonia has not been categorized as a psychogenic movement disorder for a long time, these findings once again bring the psychological component in the development of the disease into focus.

Our results, furthermore, display increased FC of the left thalamus and the right lateral premotor cortex. Even though alterations in the thalamus have not been reported in resting-state studies of task-specific dystonias, thalamus alterations are regularly observed in other forms of focal dystonia such as cervical dystonia or blepharospasm [59]. Thalamus hyperactivity has further been observed in a tapping task in WC patients [49]. Since the thalamus holds a central function in the motor circuit, being involved in sensorimotor integration and motor programming by communicating between the cortex and the basal ganglia [60], our findings are not surprising. They possibly indicate dysfunctional communication of the motor circuit in MD patients and might be an indicator for treatment methods. Recent studies investigate thalamotomy using magnetic-resonance guided focused ultrasound in MD patients with reports of first successes [61, 62]. The involvement of the lateral premotor cortex could additionally indicate interruption of the motor circuit at an early stage of movement planning. The premotor cortex receives projections from frontal cortices, conveying information through the cerebellar-thalamic loop in order to provide guidance in the planning and preparation of movements [63, 64]. The ventrolateral PM, in particular, is responsible for transforming spatial information into arm movements [65]. Studies on WC patients observed increased activation of the PMd in patients who imagined grasping a pencil with the intention of writing [66]. Furthermore, the left PMd is suggested to be important for surround inhibition, as decreased PMd inhibition was observed in right-side affected WC patients [67]. In other rsfMRI studies, mostly reduced FC with the sensorimotor cortex is reported in task-specific dystonia [30–32], contrary to our results. However, none of these studies investigated focal hand dystonia of musicians specifically. Interestingly, structural alterations in the premotor cortex have also been observed to be an identifier in the differentiation between early training or late training musicians. Musicians who started learning their instrument early show increased cortical thickness in the right premotor regions [68, 69]. As a late start of learning the instrument has repeatedly been proven to be a risk factor for musician’s dystonia [35, 36, 70], the comparably thinner premotor cortex might be a trigger for the dysfunctional communication of the premotor cortex in the motor circuit.

Our findings underline the notion that motor programs in dystonia are disrupted at an early stage, i.e., during the planning of the movement, altering the balance between excitation and inhibition in the motor network [47]. This would explain why some pianists experience dystonic cramps already as they move to the instrument with the intention of playing. They further emphasize the importance of including the movement planning phase in therapeutic approaches such as retraining.

While the cerebellum is gaining increasingly more attention in the network theory of focal dystonia, the only alterations in cerebellar FC detected in this study did not withstand correction for multiple comparisons (see supplementary material). Therefore, the reduced FC between the left Crus II of the cerebellum and the right middle temporal gyrus among MD patients is to be interpreted only as a trend. A closer inspection of the literature reveals that thus far, no resting-state FC alterations in the cerebellum have been found in musicians with focal hand dystonia [1], which would be consistent with the suggestion that the cerebellum might not be a key structure in the pathophysiology of task-specific dystonias [4]. Activation alterations were, however, found in task-based fMRI studies, revealing increased activity and effective connectivity between the cerebellum and premotor/sensorimotor cortices [17, 71], which emphasizes cerebellar involvement and the need for further research in the area.

### Functional connectivity and childhood adversity

In an attempt to unravel the mechanisms behind the often-observed neurological alterations, recent studies have examined childhood trauma and stress reactivity in MD patients and analyzed how they might affect dysfunctional movement learning. Childhood adversity has, on the one hand, been found to alter FC in emotion-related areas such as the amygdala, hippocampus, and precuneus [72], but also in areas associated with the motor circuit, such as the striatum and the cerebellum [73, 74]. Of these areas, only parts of the striatum (i.e., the globus pallidus) showed altered functional connectivity in the participating MD patients. However, the observed FC alterations were not associated with the severity of childhood adversity. Although patients assign great importance to ACEs in their individual disorder model [75]; E. Altenmüller, personal communication, spring 2020), systematic studies show that childhood trauma occurs only slightly more frequently among patients and their impact—on a neurological level—could thus far not been proven [35, 36]. Nonetheless, there is a possibility that ACEs are more associated with a form of dystonia that ranges on a psychogenic spectrum and is modulated by psychological factors such as resilience, while ACEs might not play as big a part in the pathogenesis of organic dystonia [35, 76].

### Duration of disorder

Since there is no objective measurement of symptom severity in musicians dystonia, the FC alterations could not be correlated with the severity of the disorders, as was done in similar other studies [6, 31]. Instead, we analyzed whether the duration of dystonia and the affected hand predicted the degree of FC alterations in MD patients. When correlating FC with the duration of dystonia, the studies report either no correlation [30], negative correlation with the FC of the premotor cortex and the superior parietal lobe [31], or positive correlation [32] with the negative FC of the cerebellar crus I to the right sensorimotor cortex. Our analysis did not detect an association. However, it needs to be considered that the sample size of N = 37 MD patients is relatively small for a correlation analysis [77]. Interestingly, one study that initially observed reduced FC in sensorimotor areas in WC patients found FC to increase with the severity of dystonia [31]. Further research is needed to differentiate the mechanisms behind writer’s cramp and musician's dystonia.

### Affected hand

Neuroimaging studies tend to investigate only right-handed patients to control for possible confounding effects of lateralization. However, as musicians start their training in early childhood and thereby train the hand dominantly needed for their instrument very thoroughly, the usual concept of handedness does not entirely apply to professional musicians [78, 79], which is one reason why also left-handed participants were considered in this study. Focal dystonia usually develops in the hand with the higher workload, leading to pianists being mostly affected in the right hand, while string players are more commonly affected in the left hand [80]. The previous findings that right-handed musicians are more likely to be affected in the right hand could not be confirmed in our sample, as all except for three MD patients were right-handed, but the overall sample contained more left-side affected musicians. Nonetheless, the affected hand, together with self-reported handedness, appears to be a driving factor of altered functional connectivity, as musicians affected in the right hand displayed increased FC of the left dlPFC with the right putamen as well as the left pallidum. FC in left-side affected patients was significantly lower. These findings complement previous studies where increased contralateral FC in right-handed individuals but no lateralization effects in left-handed individuals were observed at resting state [81, 82]. As in comparable studies on dystonia only patients with right-sided WC or embouchure dystonia were investigated, our findings are difficult to assess in the context of available research. In those studies, the left sensorimotor cortices of right-side affected patients were primarily investigated. However, bilateral FC alterations were observed. Our results suggest that increased functional connectivity of the left dlPFC and the bilateral pallidum, which is already present due to regular right-handedness [81], is additionally promoted by the use of the right hand at the instrument. Thus opening the question of whether lesser FC alterations suffice to predispose musician’s dystonia in the left hand. Further research is needed to clarify this question. However, acquiring considerably more left-handed patients could be difficult due to the low number of affected musicians in general.

### Limitations

As with any study using a resting-state fMRI design, there are some known methodological challenges [83]. While selecting spherical regions of interest for a seed-based analysis comes with the advantage of investigating a very specific hypothesis, it bears the risk of researchers' bias since areas might be overlooked. In our design, we decided to include entire AAL3 regions, which reduced researchers' bias in exchange for a loss of accuracy. Resting-state fMRI is furthermore very sensitive to movement artefacts, such as respiratory movement and pulse, and it is not always possible to ensure a complete “resting state” and control the level of consciousness of the participants during the scan. Apart from these methodological challenges, there are other limitations to this study.

Since the pool of eligible participants is relatively small, the inclusion criteria were liberal. This means that there was only statistical control for handedness and current medication, and these participants were not excluded. Handedness, however, is a construct that needs to be assessed differently in musicians, as discussed above. Future studies, therefore, could control more directly for the workload of the instrument together with regular handedness.

Even though our sample is large compared to similar studies, it might still not be large enough for statistical inference. The interpretation of the analyses conducted only on the sample of MD patients is especially challenging due to the small sample size. It furthermore needs to be acknowledged that all patients were treated in the same institute, which might have led to a more homogenous expression or development of the disorder. Still, to this date and to the best of our knowledge, it is the largest sample of musicians dystonia patients to have been investigated in an fMRI setting.

Lastly, the question remains whether the observed neurological alterations are predispositions or results of dystonic symptoms.

## Conclusion

In conclusion, this study offers results that are consistent with previous research. Altered functional connectivity between the putamen, pallidum, premotor cortex, and thalamus has been previously described in other task-specific dystonia studies, in resting-state as well as in task-based paradigms. We can only hypothesize that these alterations would translate to task-based functional connectivity in musicians, as it is almost impossible to realistically recreate playing an instrument during fMRI. However, similarities between rsfMRI and task-based fMRI in writer’s cramp would support this interpretation. Alterations in resting-state FC might indicate an altered flow of information between the above-named structures as well as dysfunctional neuroplastic alterations. Involvement of the premotor cortex confirms the notion that MD symptoms start in the early intentional stage of movement initiation, which was also found in writer’s cramp. The putamen and basal ganglia alterations point to deficits in inhibitory mechanisms of the motor circuit, resulting in the observed unwanted curling or extension of the fingers. Alterations in the dorsolateral prefrontal cortex have thus far been described less frequently and might indicate dysfunctional processes in movement planning and execution. In contrast to many recent studies, no altered FC between our selected seed areas and the cerebellum was detected, but this supports the findings from literature reviews that the cerebellum appears less involved in task-specific dystonia. Furthermore, contrary to our hypothesis, the observed alterations were not associated with adverse childhood experiences nor with the duration of the disorder. However, there appears to be an additive effect of right-handedness and right-side affectedness to increase functional connectivity of the dorsolateral prefrontal cortex and the basal ganglia. The study is a comparably large resting-state fMRI investigation of musicians with task-specific dystonia of the hand. The findings imply that thalamus-based interventions might be increasingly considered for treating musician’s dystonia, and that the intention of playing should be the starting point for practical treatment approaches such as re-training.

## Supplementary Information

Below is the link to the electronic supplementary material.

Supplementary file1 (PDF 76 KB)

## Data Availability

Data supporting the findings of this study are available from the corresponding author upon reasonable request.

## References

[1] Bianchi S, Fuertinger S, Huddleston H et al (2019) Functional and structural neural bases of task specificity in isolated focal dystonia: Neural bases of task specificity in dystonia. Mov Disord 34:555–563. 10.1002/mds.27649 30840778 10.1002/mds.27649 PMC6945119

[2] Altenmüller E, Jabusch H-C (2010) Focal dystonia in musicians: phenomenology, pathophysiology and triggering factors: Focal dystonia in musicians. Eur J Neurol 17:31–36. 10.1111/j.1468-1331.2010.03048.x 20590806 10.1111/j.1468-1331.2010.03048.x

[3] Battistella G, Termsarasab P, Ramdhani RA et al (2015) Isolated Focal Dystonia as a Disorder of Large-Scale Functional Networks. Cereb Cortex 27:1–13. 10.1093/cercor/bhv313 10.1093/cercor/bhv313 PMC6075177 26679193

[4] Conte A, Defazio G, Mascia M et al (2020) Advances in the pathophysiology of adult-onset focal dystonias: recent neurophysiological and neuroimaging evidence. F1000Res 9:1–11. 10.12688/f1000research.21029.2 10.12688/f1000research.21029.1 PMC6993830 32047617

[5] Corp DT, Morrison-Ham J, Jinnah HA, Joutsa J (2023) Chapter Four - The functional anatomy of dystonia: recent developments. In: Albanese A, Bhatia K, Jinnah HA (eds) International review of neurobiology. Academic Press, pp 105–136 10.1016/bs.irn.2023.04.004 37482390

[6] Hanekamp S, Simonyan K (2020) The large-scale structural connectome of task-specific focal dystonia. Hum Brain Mapp 41:3253–3265. 10.1002/hbm.25012 32311207 10.1002/hbm.25012 PMC7375103

[7] Jinnah HA, Neychev V, Hess EJ (2017) The anatomical basis for dystonia: the motor network model. Tremor Other Hyperkinet Mov (N Y) 7:506. 10.7916/D8V69X3S 29123945 10.7916/D8V69X3S PMC5673689

[8] Kita K, Rokicki J, Furuya S et al (2018) Resting-state basal ganglia network codes a motor musical skill and its disruption from dystonia: Resting-State Network in Musicians. Mov Disord 33:1472–1480. 10.1002/mds.27448 30277603 10.1002/mds.27448 PMC6220822

[9] Marapin RS, van der Horn HJ, van der Stouwe AM et al (2022) Altered brain connectivity in hyperkinetic movement disorders: a review of resting-state fMRI. NeuroImage Clin 37:103302. 10.1016/j.nicl.2022.103302 36669351 10.1016/j.nicl.2022.103302 PMC9868884

[10] Neychev VK, Gross R, Lehéricy S et al (2011) The functional neuroanatomy of dystonia. Neurobiol Dis 42:185–201. 10.1016/j.nbd.2011.01.026 21303695 10.1016/j.nbd.2011.01.026 PMC3478782

[11] Candia V, Wienbruch C, Elbert T et al (2003) Effective behavioral treatment of focal hand dystonia in musicians alters somatosensory cortical organization. Proc Natl Acad Sci 100:7942–7946. 10.1073/pnas.1231193100 12771383 10.1073/pnas.1231193100 PMC164692

[12] Elbert T, Candia V, Altenmüller E et al (1998) Alteration of digital representations in somatosensory cortex in focal hand dystonia. NeuroReport 9:3571–3575. 10.1097/00001756-199811160-00006 9858362 10.1097/00001756-199811160-00006

[13] Sadnicka A, Wiestler T, Butler K et al (2023) Intact finger representation within primary sensorimotor cortex of musician’s dystonia. Brain 146:1511–1522. 10.1093/brain/awac356 36170332 10.1093/brain/awac356 PMC10115231

[14] Quartarone A, Hallett M (2013) Emerging concepts in the physiological basis of dystonia. Mov Disord 28:958–967. 10.1002/mds.25532 23893452 10.1002/mds.25532 PMC4159671

[15] Simonyan K, Cho H, Hamzehei Sichani A et al (2017) The direct basal ganglia pathway is hyperfunctional in focal dystonia. Brain 140:3179–3190. 10.1093/brain/awx263 29087445 10.1093/brain/awx263 PMC5841143

[16] Filip P, Lungu OV, Bareš M (2013) Dystonia and the cerebellum: a new field of interest in movement disorders? Clin Neurophysiol 124:1269–1276. 10.1016/j.clinph.2013.01.003 23422326 10.1016/j.clinph.2013.01.003

[17] Kita K, Furuya S, Osu R et al (2021) Aberrant cerebello-cortical connectivity in pianists with focal task-specific dystonia. Cereb Cortex 31:4853–4863. 10.1093/cercor/bhab127 34013319 10.1093/cercor/bhab127

[18] Preibisch C, Berg D, Hofmann E et al (2001) Cerebral activation patterns in patients with writer’s cramp: a functional magnetic resonance imaging study. J Neurol 248:10–17. 10.1007/s004150170263 11266013 10.1007/s004150170263

[19] Sadnicka A, Hoffland BS, Bhatia KP et al (2012) The cerebellum in dystonia – help or hindrance? Clin Neurophysiol 123:65–70. 10.1016/j.clinph.2011.04.027 22078259 10.1016/j.clinph.2011.04.027

[20] Lanciego JL, Luquin N, Obeso JA (2012) Functional neuroanatomy of the basal ganglia. Cold Spring Harb Perspect Med 2:a009621. 10.1101/cshperspect.a009621 23071379 10.1101/cshperspect.a009621 PMC3543080

[21] Doyon J, Penhune V, Ungerleider LG (2003) Distinct contribution of the cortico-striatal and cortico-cerebellar systems to motor skill learning. Neuropsychologia 41:252–262. 10.1016/S0028-3932(02)00158-6 12457751 10.1016/s0028-3932(02)00158-6

[22] Mittal VA, Bernard JA, Walther S (2021) Cerebellar-thalamic circuits play a critical role in psychomotor function. Mol Psychiatry 26:3666–3668. 10.1038/s41380-020-00935-9 33203993 10.1038/s41380-020-00935-9 PMC8126567

[23] Fuertinger S, Simonyan K (2018) Task-specificity in focal dystonia is shaped by aberrant diversity of a functional network kernel: abnormal network kernel in focal dystonia. Mov Disord 33:1918–1927. 10.1002/mds.97 30264427 10.1002/mds.97 PMC6309584

[24] Konczak J, Abbruzzese G (2013) Focal dystonia in musicians: linking motor symptoms to somatosensory dysfunction. Front Hum Neurosci. 10.3389/fnhum.2013.00297 23805090 10.3389/fnhum.2013.00297 PMC3691509

[25] Biswal B, Yetkin FZ, Haughton VM, Hyde JS (1995) Functional connectivity in the motor cortex of resting human brain using echo-planar MRI. Magn Reson Med 34:537–541. 10.1002/mrm.1910340409 8524021 10.1002/mrm.1910340409

[26] Fox MD, Raichle ME (2007) Spontaneous fluctuations in brain activity observed with functional magnetic resonance imaging. Nat Rev Neurosci 8:700–711. 10.1038/nrn2201 17704812 10.1038/nrn2201

[27] Mohammadi B, Kollewe K, Samii A et al (2012) Changes in resting-state brain networks in writer’s cramp. Hum Brain Mapp 33:840. 10.1002/hbm.21250 21484954 10.1002/hbm.21250 PMC6870480

[28] Mantel T, Meindl T, Li Y et al (2018) Network-specific resting-state connectivity changes in the premotor-parietal axis in writer’s cramp. NeuroImage Clin 17:137–144. 10.1016/j.nicl.2017.10.001 29085775 10.1016/j.nicl.2017.10.001 PMC5650679

[29] Ramdhani RA, Kumar V, Velickovic M et al (2014) What’s special about task in dystonia? A voxel-based morphometry and diffusion weighted imaging study. Mov Disord 29:1141–1150. 10.1002/mds.25934 24925463 10.1002/mds.25934 PMC4139455

[30] Haslinger B, Noé J, Altenmüller E et al (2017) Changes in resting-state connectivity in musicians with embouchure dystonia. Mov Disord 32:450–458. 10.1002/mds.26893 27911020 10.1002/mds.26893

[31] Dresel C, Li Y, Wilzeck V et al (2014) Multiple changes of functional connectivity between sensorimotor areas in focal hand dystonia. J Neurol Neurosurg Psychiatry 85:1245–1252. 10.1136/jnnp-2013-307127 24706945 10.1136/jnnp-2013-307127

[32] Delnooz CCS, Helmich RC, Toni I, van de Warrenburg BPC (2012) Reduced parietal connectivity with a premotor writing area in writer’s cramp. Mov Disord 27:1425–1431. 10.1002/mds.25029 22886735 10.1002/mds.25029

[33] Rosenkranz K (2005) Pathophysiological differences between musician’s dystonia and writer’s cramp. Brain 128:918–931. 10.1093/brain/awh402 15677703 10.1093/brain/awh402

[34] Detari A, Clark T, Egermann H (2022) Musician’s Focal Dystonia: A mere neurological disorder? The role of non-organic factors in the onset of Musician’s Focal Dystonia: an exploratory Grounded Theory study. J Music Health Wellbeing 1–15

[35] Alpheis S, Altenmüller E, Scholz DS (2022) Influence of adverse childhood experiences and perfectionism on musician’s dystonia: a case control study. Tremor Other Hyperkinetic Mov 12:1–15. 10.5334/tohm.687 10.5334/tohm.687 PMC8932351 35415008

[36] Alpheis S, Sinke C, Burek J et al (2024) Stress in musicians with and without focal dystonia is not reflected in limbic circuit activation. Mov Disord 39:2075–2086. 10.1002/mds.29941 39077793 10.1002/mds.29941

[37] Holz NE, Berhe O, Sacu S et al (2023) Early social adversity, altered brain functional connectivity, and mental health. Biol Psychiatry 93:430–441. 10.1016/j.biopsych.2022.10.019 36581495 10.1016/j.biopsych.2022.10.019

[38] Dolfen N, King BR, Schwabe L et al (2021) Stress modulates the balance between hippocampal and motor networks during motor memory processing. Cereb Cortex 31:1365–1382. 10.1093/cercor/bhaa302 33106842 10.1093/cercor/bhaa302

[39] Corr R, Glier S, Bizzell J et al (2022) Stress-related hippocampus activation mediates the association between polyvictimization and trait anxiety in adolescents. Soc Cogn Affect Neurosci 17:767–776. 10.1093/scan/nsab129 34850948 10.1093/scan/nsab129 PMC9340110

[40] Bernstein DP, Stein JA, Newcomb MD et al (2003) Development and validation of a brief screening version of the Childhood Trauma Questionnaire. Child Abuse Negl 27:169–190. 10.1016/S0145-2134(02)00541-0 12615092 10.1016/s0145-2134(02)00541-0

[41] Wingenfeld K, Spitzer C, Mensebach C et al (2010) Die deutsche version des childhood trauma questionnaire (CTQ): Erste Befunde zu den psychometrischen Kennwerten. Psychother Psych Med 60:442–450. 10.1055/s-0030-1247564 10.1055/s-0030-1247564 20200804

[42] Friston KJ, Williams S, Howard R et al (1996) Movement-Related effects in fMRI time-series. Magn Reson Med 35:346–355. 10.1002/mrm.1910350312 8699946 10.1002/mrm.1910350312

[43] Power JD, Barnes KA, Snyder AZ et al (2012) Spurious but systematic correlations in functional connectivity MRI networks arise from subject motion. Neuroimage 59:2142–2154. 10.1016/j.neuroimage.2011.10.018 22019881 10.1016/j.neuroimage.2011.10.018 PMC3254728

[44] Rolls ET, Huang C-C, Lin C-P et al (2020) Automated anatomical labelling atlas 3. Neuroimage 206:116189. 10.1016/j.neuroimage.2019.116189 31521825 10.1016/j.neuroimage.2019.116189

[45] Tzourio-Mazoyer N, Landeau B, Papathanassiou D et al (2002) Automated anatomical labeling of activations in SPM using a macroscopic anatomical parcellation of the MNI MRI single-subject brain. Neuroimage 15:273–289. 10.1006/nimg.2001.0978 11771995 10.1006/nimg.2001.0978

[46] Mayka MA, Corcos DM, Leurgans SE, Vaillancourt DE (2006) Three-dimensional locations and boundaries of motor and premotor cortices as defined by functional brain imaging: a meta-analysis. Neuroimage 31:1453–1474. 10.1016/j.neuroimage.2006.02.004 16571375 10.1016/j.neuroimage.2006.02.004 PMC2034289

[47] Battistella G, Simonyan K (2019) Top-down alteration of functional connectivity within the sensorimotor network in focal dystonia. Neurology 92:e1843–e1851. 10.1212/WNL.0000000000007317 30918091 10.1212/WNL.0000000000007317 PMC6550502

[48] Rothkirch I, Granert O, Knutzen A et al (2018) Dynamic causal modeling revealed dysfunctional effective connectivity in both, the cortico-basal-ganglia and the cerebello-cortical motor network in writers’ cramp. Neuroimage Clin 18:149–159. 10.1016/j.nicl.2018.01.015 29868443 10.1016/j.nicl.2018.01.015 PMC5984595

[49] Peller M (2006) The basal ganglia are hyperactive during the discrimination of tactile stimuli in writer’s cramp. Brain 129:2697–2708. 10.1093/brain/awl181 16854945 10.1093/brain/awl181

[50] Gerfen CR, Engber TM, Mahan LC et al (1990) D1 and D2 dopamine receptor-regulated gene expression of striatonigral and striatopallidal neurons. Science 250:1429–1432. 10.1126/science.2147780 2147780 10.1126/science.2147780

[51] Ip CW, Isaias IU, Kusche-Tekin BB et al (2016) Tor1a+/- mice develop dystonia-like movements via a striatal dopaminergic dysregulation triggered by peripheral nerve injury. Acta Neuropathol Commun 4:108. 10.1186/s40478-016-0375-7 27716431 10.1186/s40478-016-0375-7 PMC5048687

[52] Draganski B, Kherif F, Klöppel S et al (2008) Evidence for Segregated and Integrative Connectivity Patterns in the Human Basal Ganglia. J Neurosci 28:7143–7152. 10.1523/JNEUROSCI.1486-08.2008 18614684 10.1523/JNEUROSCI.1486-08.2008 PMC6670486

[53] Halsband U, Lange RK (2006) Motor learning in man: a review of functional and clinical studies. J Physiol-Paris 99:414–424. 10.1016/j.jphysparis.2006.03.007 16730432 10.1016/j.jphysparis.2006.03.007

[54] Taniguchi S, Kajiyama Y, Kochiyama T et al (2024) New insights into freezing of gait in Parkinson’s disease from spectral dynamic causal modeling. Mov Disord 39:1982–1992. 10.1002/mds.29988 39295169 10.1002/mds.29988

[55] Ceballos-Baumann AO, Passingham RE, Warner T et al (1995) Overactive prefrontal and underactive motor cortical areas in idiopathic dystonia. Ann Neurol 37:363–372. 10.1002/ana.410370313 7695236 10.1002/ana.410370313

[56] Nowak DA, Fink GR (2009) Psychogenic movement disorders: aetiology, phenomenology, neuroanatomical correlates and therapeutic approaches. Neuroimage 47:1015–1025. 10.1016/j.neuroimage.2009.04.082 19426818 10.1016/j.neuroimage.2009.04.082

[57] Vuilleumier P (2005) Hysterical conversion and brain function. In: Laureys S (ed) Progress in brain research. Elsevier, pp 309–329 10.1016/S0079-6123(05)50023-2 16186033

[58] Schrag AE, Mehta AR, Bhatia KP et al (2013) The functional neuroimaging correlates of psychogenic versus organic dystonia. Brain 136:770–781. 10.1093/brain/awt008 23436503 10.1093/brain/awt008 PMC3580272

[59] Giannì C, Pasqua G, Ferrazzano G et al (2022) Focal dystonia. Neurology 98:e1499–e1509. 10.1212/WNL.0000000000200022 35169015 10.1212/WNL.0000000000200022

[60] Herrero M-T, Barcia C, Navarro J (2002) Functional anatomy of thalamus and basal Ganglia. Child’s Nervous Syst 18:386–404. 10.1007/s00381-002-0604-1 10.1007/s00381-002-0604-1 12192499

[61] Horisawa S, Yamaguchi T, Abe K et al (2021) Magnetic resonance-guided focused ultrasound thalamotomy for focal hand dystonia: a pilot study. Mov Disord 36:1955–1959. 10.1002/mds.28613 34050695 10.1002/mds.28613 PMC8453941

[62] Maamary J, Peters J, Kyle K et al (2023) Evaluation of the efficacy and safety of MRI-guided focused ultrasound (MRgFUS) for focal hand dystonia: study protocol for an open-label non-randomised clinical trial. BMJ Neurol Open 5:e000522. 10.1136/bmjno-2023-000522 37900622 10.1136/bmjno-2023-000522 PMC10603452

[63] Thompson AE, Thompson PD (2023) Chapter 22 - Frontal lobe motor syndromes. In: Younger DS (ed) Handbook of clinical neurology. Elsevier, pp 443–455 10.1016/B978-0-323-98817-9.00008-9 37620084

[64] Wise SP (1985) The primate premotor cortex: past, present, and preparatory. Annu Rev Neurosci 8:1–19. 10.1146/annurev.ne.08.030185.000245 3920943 10.1146/annurev.ne.08.030185.000245

[65] Rizzolatti G, Fogassi L, Gallese V (2002) Motor and cognitive functions of the ventral premotor cortex. Curr Opin Neurobiol 12:149–154. 10.1016/s0959-4388(02)00308-2 12015230 10.1016/s0959-4388(02)00308-2

[66] Delnooz CCS, Helmich RC, Medendorp WP et al (2011) Writer’s cramp: Increased dorsal premotor activity during intended writing. Hum Brain Mapp 34:613–625. 10.1002/hbm.21464 22113948 10.1002/hbm.21464 PMC6870150

[67] Beck S, Houdayer E, Pirio Richardson S, Hallett M (2009) The role of inhibition from the left dorsal premotor cortex in right-sided focal hand dystonia. Brain Stimul 2:208–214. 10.1016/j.brs.2009.03.004 20633420 10.1016/j.brs.2009.03.004 PMC3787900

[68] Bailey JA, Zatorre RJ, Penhune VB (2014) Early musical training is linked to gray matter structure in the ventral premotor cortex and auditory–motor rhythm synchronization performance. J Cogn Neurosci 26:755–767. 10.1162/jocn_a_00527 24236696 10.1162/jocn_a_00527

[69] Shenker JJ, Steele CJ, Zatorre RJ, Penhune VB (2023) Using cortico-cerebellar structural patterns to classify early- and late-trained musicians. Hum Brain Mapp 44:4512–4522. 10.1002/hbm.26395 37326147 10.1002/hbm.26395 PMC10365229

[70] Schmidt A, Jabusch H-C, Altenmüller E et al (2013) Challenges of making music: what causes musician’s dystonia? JAMA Neurol 70:1456. 10.1001/jamaneurol.2013.3931 24217453 10.1001/jamaneurol.2013.3931

[71] Kadota H, Nakajima Y, Miyazaki M et al (2010) An fMRI study of musicians with focal dystonia during tapping tasks. J Neurol 257:1092–1098. 10.1007/s00415-010-5468-9 20143109 10.1007/s00415-010-5468-9

[72] Fan J, Gao F, Wang X et al (2023) Right amygdala–right precuneus connectivity is associated with childhood trauma in major depression patients and healthy controls. Soc Cognit Affect Neurosci 18:64. 10.1093/scan/nsac064 10.1093/scan/nsac064 PMC10036873 36639930

[73] Keator DB, Salgado F, Madigan C et al (2024) Adverse childhood experiences, brain function, and psychiatric diagnoses in a large adult clinical cohort. Front Psychiatry 15:1401745. 10.3389/fpsyt.2024.1401745 39469474 10.3389/fpsyt.2024.1401745 PMC11513356

[74] Mundorf A, Merklein SA, Rice LC et al (2024) Early adversity affects cerebellar structure and function-a systematic review of human and animal studies. Dev Psychobiol 66:e22556. 10.1002/dev.22556 39378310 10.1002/dev.22556

[75] Schneider J, Scholz D, Altenmüller E (2021) Impact of psychic traumatization on the development of musicians’ dystonia - six exploratory case studies. Med Probl Perform Artist 36:1–9 10.21091/mppa.2021.1001 33647091

[76] Quartarone A, Rizzo V, Terranova C et al (2009) Abnormal sensorimotor plasticity in organic but not in psychogenic dystonia. Brain 132:2871–2877. 10.1093/brain/awp213 19690095 10.1093/brain/awp213 PMC2997979

[77] Bujang MA (2024) An elaboration on sample size determination for correlations based on effect sizes and confidence interval width: a guide for researchers. Restor Dent Endod 49:e21. 10.5395/rde.2024.49.e21 38841381 10.5395/rde.2024.49.e21 PMC11148401

[78] Jäncke L, Schlaug G, Steinmetz H (1997) Hand skill asymmetry in professional musicians. Brain Cogn 34:424–432. 10.1006/brcg.1997.0922 9292190 10.1006/brcg.1997.0922

[79] Schlaug G, Jäncke L, Huang Y et al (1995) Increased corpus callosum size in musicians. Neuropsychologia 33:1047–1055. 10.1016/0028-3932(95)00045-5 8524453 10.1016/0028-3932(95)00045-5

[80] Jabusch H-C, Altenmüller E (2006) Focal dystonia in musicians: From phenomenology to therapy. Adv Cogn Psychol 2:207–220. 10.2478/v10053-008-0056-6

[81] Tejavibulya L, Peterson H, Greene A et al (2022) Large-scale differences in functional organization of left- and right-handed individuals using whole-brain, data-driven analysis of connectivity. Neuroimage 252:119040. 10.1016/j.neuroimage.2022.119040 35272202 10.1016/j.neuroimage.2022.119040 PMC9013515

[82] Pool E-M, Rehme AK, Eickhoff SB et al (2015) Functional resting-state connectivity of the human motor network: differences between right- and left-handers. Neuroimage 109:298–306. 10.1016/j.neuroimage.2015.01.034 25613438 10.1016/j.neuroimage.2015.01.034 PMC4981638

[83] Cole DM, Smith SM, Beckmann CF (2010) Advances and pitfalls in the analysis and interpretation of resting-state FMRI data. Front Syst Neurosci 4:8. 10.3389/fnsys.2010.00008 20407579 10.3389/fnsys.2010.00008 PMC2854531

