# Supplementary material for: Increased functional connectivity of motor regions and dorsolateral prefrontal cortex in musicians with focal hand dystonia
Source: J Neurol. 2025 Mar 22;272(4):281. doi: 10.1007/s00415-025-13018-y (PMC11929630; doi:10.1007/s00415-025-13018-y)
Supplement: Supplementary file 1 — Supplementary file1 (PDF 76 KB) [file 415_2025_13018_MOESM1_ESM.pdf]

Increased Functional Connectivity of Motor Regions and Dorsolateral Prefrontal Cortex in Musicians with Focal Hand Dystonia

Journal of Neurology

Stine Alpheis, MSc, Christopher Sinke, PhD, Julian Burek, Tillmann H. C. Krüger, MD, Eckart Altenmüller, MD and Daniel S. Scholz, PhD

Corresponding author: Daniel S. Scholz, Musikhochschule Lübeck, Germany
m: daniel.scholz@mh-luebeck.de

Supplementary Table

Seed ROI	MNI					
Target Regions	x	y	z	Cluster size k	t value	p-value
MD > HM						
Frontal Sup 2 R						
Putamen L	-24	2	4	125	5.04	.006
Frontal Inf Oper R						
Frontal Sup Medial R	4	40	36	145	4.45	.004
Olfactory L						
Frontal Inf Oper R	50	10	0	136	4.55	.005
Angular L						
Pallidum L	-20	2	2	87	4.46	.040
Putamen R						
Cingulate Mid R	4	42	30	95	4.67	.034
Thalamus AV L						
Frontal Mid 2 R	38	14	44	122	4.41	.010
Thalamus AV R						
Putamen L	-24	2	2	121	5.39	.012
Thal MDm L						
Frontal Inf Tri R	56	28	22	136	4.66	.007
Thal MDl L						
Frontal Mid 2 R	42	8	38	162	4.36	.002
HM > MD						
Temporal Pole Mid R						
Cerebellum Crus 2 L	-40	-64	-40	90	4.16	.046

Note: Results are presented FWE_{corr} at cluster level $p < 0.05$. Significant clusters were identified based on the AAL3 in MRIcroGL. Abbreviations: ROI, Regions of Interest; L, left; R, right; dlPFC, dorsolateral prefrontal cortex; MD, musician's dystonia patients; HM, healthy musicians; MNI, montreal national institute of health coordinates; k, number of voxels in the significant cluster.
